# Intravesical Instillation of Mitomycin C: A Cause of Delayed Bladder Perforation?

**DOI:** 10.1155/2012/576519

**Published:** 2012-12-23

**Authors:** Marta Penna, Kiki Mistry, Pallavi Pal, Chitale Sudhanshu

**Affiliations:** Department of Urology, Whittington Health, Magdala Avenue, London N19 5NF, UK

## Abstract

We present a case of bladder perforation secondary to intravesical instillation of mitomycin C following transurethral resection of bladder tumour (TURBT) and the role of early detection leading to successful conservative management. We also review the key relevant literature.

## 1. Introduction

The European Association of Urology and American Urological Association guidelines recommend immediate postoperative instillation of intravesical chemotherapy in cases of nonmuscle invasive bladder tumour (NMIBT). There is strong evidence showing immediate postoperative instillation of chemotherapy reduces the risk of recurrence by 39% [1]. 

Mitomycin C (MMC) instillation is routinely administered and generally safe. However, several papers have reported an association between postoperative intravesical MMC instillation and bladder perforation leading to severe morbidity including death. We present a case of bladder perforation suspected mostly to be secondary to MMC instillation post-TURBT and a review of the current literature.

## 2. Case Report

A 77-year-old Caucasian gentleman underwent TURBT for a 1 cm recurrent superficial papillary tumour located high on the posterior wall, at its junction with the dome of the bladder. The primary tumour was initially resected 17 years previously showing a G1 pTa transitional cell carcinoma (TCC) but no subsequent surveillance had been organised as the patient was lost for follow up. The patient was an ex-smoker, stopping 20 years ago with a 20 pack year history. His past medical history had open bilateral inguinal hernia repair but no significant family or drug history.

The lesion was completely resected and deep muscle biopsy taken separately using cold cup biopsy forceps and the biopsy site diathermised. Intraoperatively, there was no endoscopic evidence of bladder wall perforation; hence mitomycin C, 40 mg in 40 cc of water for injection, was instilled into the bladder postoperatively. Histology revealed a 30 mm Grade 1/2 (low grade) pTa papillary TCC.

Following removal of catheter, delayed to the second postoperative day due to haematuria, the patient failed to void for the first eight hours. He subsequently developed excruciating pain in the lower abdomen on attempts at voiding, leading to a vasovagal syncopal attack. On examination there was evidence of peritonism localized to the suprapubic region. He was found to be in urinary retention and thus re-catheterised.

**Figure 1:**
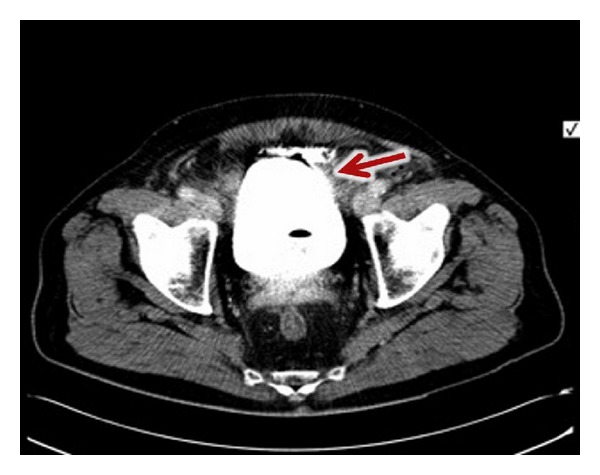
Computed tomography with retrograde cystography (axial view) showing extraperitoneal bladder perforation along anterior wall (arrow points to extravasated contrast).

Computed tomography (CT) with retrograde cystography demonstrated localised extraperitoneal extravasation in continuity with the anterior bladder wall (Figure 1) with no intraperitoneal spillage. The patient was managed conservatively with analgesia, antibiotics and a 16 F indwelling catheter for 4 weeks. At 2 weeks follow up nontender suprapubic induration was palpable which resolved at 6 weeks.

**Figure 2:**
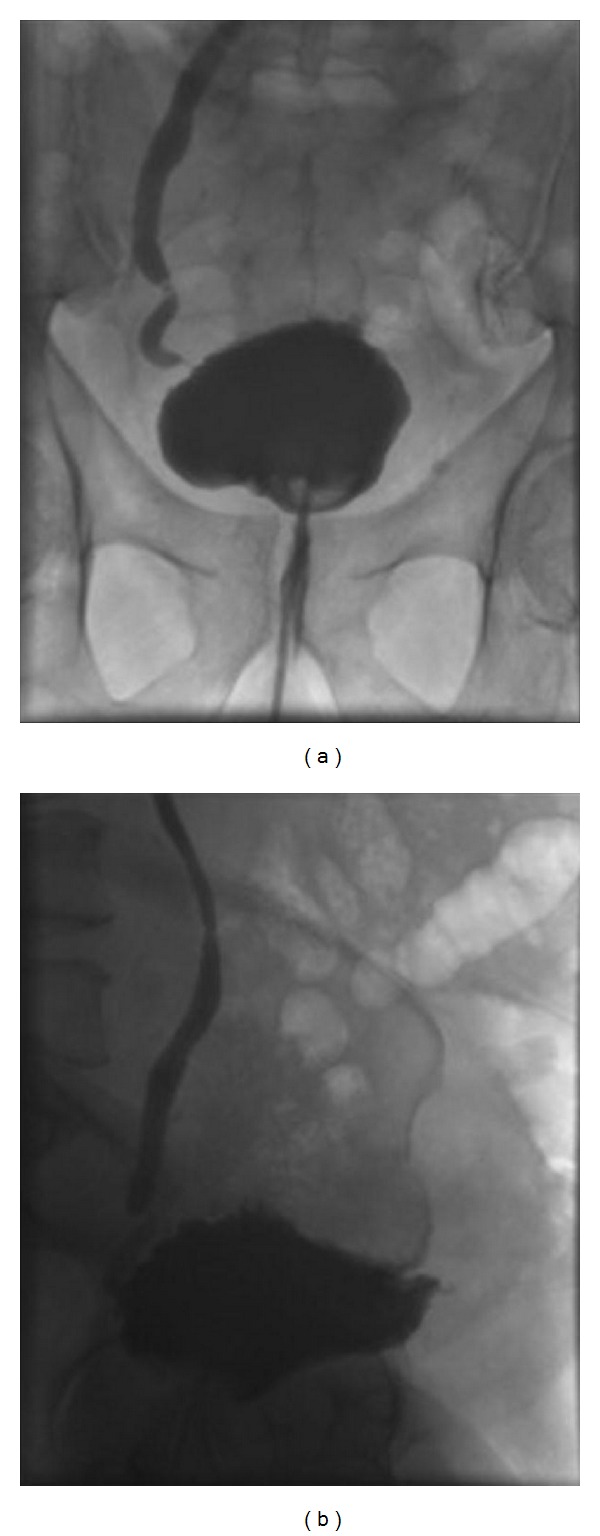
Follow-up cystography showing complete healing of bladder perforation following conservative management: (a) anteroposterior view, (b) lateral view.

Cystogram at 4 weeks showed no evidence of urinary leak (Figure 2). The urinary catheter was removed with no further complications.

## 3. Discussion

Transurethral resection of bladder tumours followed by perioperative instillation of a chemotherapeutic agent is currently the gold standard treatment for NMIBT. Small and clinically harmless bladder perforations have been detected on cystography in up to 58% of patients post-TURBT [2]. However, in the presence of endoscopic evidence of perforation intravesical instillation of a chemotherapeutic agent is generally not recommended. 

Golan et al. conducted a retrospective observational study to examine the clinical characteristics and long-term outcomes of patients with post-TURBT perforations requiring surgical repair [3]. The study included 4144 patients with a median age of 77 years and 80% of patients having Ta or T1 bladder cancer. Fifteen patients (0.36%) required open surgical repair. The greatest risk factors reported were increasing age, large posterior wall tumours, and heavily pretreated bladders. In fact, 87% of the 15 patients with bladder perforation had had a previous TURBT and 93% had had previous intravesical instillations. Two out of the 15 patients had previous radiotherapy following TURBT. The diagnosis of bladder perforation was made on average 6.1 days post-op. Two out of the 15 patients undergoing surgery died of septic shock and multiorgan failure; in both cases diagnosis of perforation was delayed, highlighting the crucial need for increased awareness and early detection of such a complication. Importantly, Golan's study showed an overall low likelihood of extravesical tumour seeding despite bladder perforation and open surgical repair. 

Although perioperative intravesical instillation is a routine, safe and effective therapeutic modality, a number of case reports of bladder perforation secondary to this adjuvant treatment have been published in the literature. Doherty et al. observed that more extensive bladder wall and fat necrosis of extravesical tissue occurred when MMC instillation was administered than in that seen following TURBT alone [4]. More recently, Lim et al. published a case report of a 79 year old with recurrent pTaG2 bladder cancer who developed bladder perforation post MMC instillation [5]. Severe systemic toxicity occurred requiring intensive care after surgical repair. Exploratory laparotomy revealed a large necrotic defect in the anterior bladder wall and a retropubic abscess. Lim suggested that the secondary bladder perforation could be the result of an area of attenuated muscularis propria leading to necrosis exacerbated by MMC instillation. The author also mentioned that the patient's preexisting peripheral vascular disease and thus poor tissue oxygenation may have impeded adequate bladder healing. 

Instillation of other chemotherapeutic agents, including epirubicin, also appears to be associated with bladder perforations [6]. It was postulated that delayed perforation may occur secondary to cytotoxic effects on the bladder tissue of the instilled drugs leading to extensive paravesical fibrotic reaction and necrosis with a significantly reduced bladder capacity. The perforation was repaired surgically with uneventful recovery. 

Functional changes in the bladder after intravesical administration of mitomycin C or epirubicin have been studied in rats. Michielson et al. measured changes in vesical capacity and bladder wall compliance (defined as change in vesical volume induced by a given change in pressure) in rats after intravesical instillations of mitomycin C, epirubicin, or saline, and a control group with no instillations [7]. Drug doses administered reflected those used in clinical therapeutic practice. Results showed that weekly instillations of mitomycin C, and to a lesser degree with epirubicin, caused a statistically significant reduction in both vesical volume and compliance, which persisted for up to 3 weeks post cessation of instillations. Similar transient effects on bladder function with recovery to baseline after an average of 3 weeks was also noted by Post et al., with significantly less damage from doxorubicin compared to mitomycin C [8].

Although MMC instillation after TURBT is a well established treatment regime, current literature and our case report highlight the need for urologists to be aware and vigilant of the increased risk of bladder perforation posed by chemotherapeutic agents. As highlighted in our case, prolonged period of urinary retention (bladder over-distension) particularly in the elderly with existent outflow obstruction, subsequent to post-TURBT catheter removal, could further compromise the resection site already weakened by the chemotherapeutic agent thereby precipitating bladder wall perforation. Conservative management is safe and remains feasible with early detection and confirmation of an extraperitoneal perforation as in present case. 

Intraperitoneal perforations (irrespective of size) would generally warrant surgical exploration and repair if detected at the time of TURBT. Delayed diagnosis would lead to increased morbidity and even mortality [2].

Further larger prospective studies are required to investigate the exact mechanism of MMC induced cytotoxicity leading to bladder wall perforation and long-term clinical and oncological outcomes following bladder perforation.
